# Recombinant Human Interferon Alpha 2b Prevents and Reverses Experimental Pulmonary Hypertension

**DOI:** 10.1371/journal.pone.0096720

**Published:** 2014-05-16

**Authors:** Eileen M. Bauer, Han Zheng, Michael T. Lotze, Philip M. Bauer

**Affiliations:** 1 Department of Surgery, University of Pittsburgh School of Medicine, Pittsburgh, Pennsylvania, United States of America; 2 University of Pittsburgh Cancer Institute, University of Pittsburgh School of Medicine, Pittsburgh, Pennsylvania, United States of America; 3 Department of Pharmacology and Chemical Biology, University of Pittsburgh School of Medicine, Pittsburgh, Pennsylvania, United States of America; 4 Vascular Medicine Institute, University of Pittsburgh School of Medicine, Pittsburgh, Pennsylvania, United States of America; University of Illinois College of Medicine, United States of America

## Abstract

Pulmonary hypertension (PH) is a progressive and fatal disease with no cure. Vascular remodeling in PH involves intraluminal growth of endothelial and smooth muscle cells, leading to obliterative vascular lesions. Cell growth in these lesions is quasi-neoplastic, with evidence of monoclonality, apoptosis resistance and cancer-like metabolic derangements. Herein we tested the effect of human interferon alpha 2b (IFNα), a pleiotropic cytokine and anti-cancer therapeutic, on the development and progression of PH in the rat SU5416/hypoxia (SUH) model and mouse hypoxia model of the disease. In both models IFNα attenuated the development of PH and reversed established PH as assessed by measuring right ventricular systolic pressure and right ventricular hypertrophy. The effect of IFNα was dependent on the type I interferon receptor (IFNAR) since mice lacking a subunit of the IFNAR were not protected by IFNα. Morphometric analysis of pulmonary aterioles from hypoxic mice or SUH rats showed that IFNα inhibited pulmonary vascular remodeling in both models and that IFNα reversed remodeling in SUH rats with established disease. Immunohistochemical staining revealed that IFNα decreased the number of PCNA and Tunel positive cells in the wall of pulmonary arterioles. *In vitro*, IFNα inhibited proliferation of human pulmonary artery smooth muscle cells and as well as human pulmonary artery endothelial cell proliferation and apoptosis. Together these findings demonstrate that IFNα reverses established experimental PH and provide a rationale for further exploration of the use of IFNα and other immunotherpies in PH.

## Introduction

Pulmonary Hypertension (PH) is a devastating disease characterized by increased pulmonary artery pressure, right ventricular (RV) failure and death. Although the natural history of the disease is incompletely understood, the traditional view is that endothelial dysfunction and upregulation of pulmonary vasoconstrictors leads to pulmonary vasoconstriction and increased pulmonary artery (PA) pressure. In addition, several pulmonary vasoconstrictors are also smooth muscle cell (SMC) mitogens [1] and prolonged exposure to these vasoconstrictors results in hypertrophy and proliferation of medial SMC [2].

In severe disease the PAs of PH patients exhibit invasive growth of endothelial cells (EC) into the vessel lumen resulting in luminal obstruction by clusters of ECs known as plexiform lesions. EC growth in plexiform lesions is aberrant with some areas containing a solid core of ECs and others exhibiting various stages of angiogenesis [3]. In addition, there is evidence of EC monoclonality [4], resistance of ECs to apoptosis [5], and a cancer-like shift to glycolysis [6] within plexiform lesions. Thus, the vascular lesions in PH exhibit several hallmarks of cancer [7]. These findings represent a major paradigm shift in PH research, which has relied on models of hypoxic vasoconstriction, and indicate that concepts derived from the cancer field should be considered when developing PH therapeutics [4].

Type I interferons (IFN), were identified in 1957 by Isaacs and Lindenmann based on the ability to inhibit viral replication [8], [9]. The type I IFN family of at least 15 subtypes includes the IFNα family of 13 functional subtypes of IFNα, IFN-β, and IFNω [10]. The individual IFNα subtypes share the same receptor and exhibit similar biological activities [10]. Type I interferons exhibit a variety of biological effects in addition to those on viral replication, including antitumor activity, anti-angiogenic activity, and utility in multiple sclerosis [11]. Today, IFNα is the most widely used therapeutic cytokine in patients.

Little is known about the effect of IFNα on the pathogenesis of pulmonary hypertension. There are case studies of patients receiving IFNα therapy for the treatment of hepatitis C or chronic myelogenous leukemia developing reversible or irreversible PH [12]–[14]. On the other hand IFNα has been used to treat PH associated with pulmonary capillary hemangiomatosis [15], [16]. In several instances IFNα stabilized or caused regression of pulmonary capillary hemangiomatosis associated PH.

The goal of the present study was to evaluate the effect of IFNα on experimental PH. Based on the case studies demonstrating IFNα-induced PH, and our data showing activation of interferon response factor-3 in PH our original hypothesis was that IFNα would exacerbate experimental PH. Instead, we found that, in both the mouse model of chronic hypoxia and the rat model of SU5416 plus chronic hypoxia, IFNα not only attenuated the development of PH, but also reversed established disease.

## Methods

Human IPAH cells and serum samples were obtained in compliance with University of Pittsburgh Institutional Review Board (IRB) guidelines **and the studies were approved by the University of Pittsburgh IRB. All** patients gave written consent. Animal studies were approved by the University of Pittsburgh Institutional Animal Care and Use Committee (University of Pittsburgh Animal Assurance # A3187–01).

### Animal Use

C57BL/6J mice were purchased from The Jackson Laboratory (Bar Harbor, ME) and 129S6/SvEvTac WT mice were purchased from Taconic Farms (Watertown, NY). IFNAR-deficient mice (IFN-IR−/−129S6) were a kind gift of Akiko Iwasaki (Yale University, New Haven, CT) [17]. Age-matched 8- to 12-wk-old male mice were used for the studies. 225–250 g Sprague Dawley rats purchased from Charles Rivers were used for the studies.

### Chronic Hypoxia Mouse Model

Eight to ten week old male mice were placed into a partially ventilated Plexiglas chamber (Biospherix,) and exposed to chronic hypoxia (FIO2 = 0.10, 90% nitrogen) for 21 or 42 days under normobaric conditions. Mice maintained in room air served as normoxic controls. For all mouse studies mice were treated with daily subcutaneous injections of 10^4^ IU human recombinant interferon alpha 2b (Intron A; Schering Corporation, Kenilworth, NJ). The dose of interferon alpha 2b was chosen based on a search of the literature [18], [19]. The interferon was reconstituted using sterile water for injection, USP provided by the manufacturer and was stored at 4°C after reconstitution per the manufacturer's instructions.

### Rat SU5416/Hypoxia Model

225–250 g male Sprague Dawley Rats were injected with a single dose of 20 mg/kg s.c. SU-5416 (A VEGF receptor inhibitor) or vehicle and were placed into a partially ventilated Plexiglas chamber (Biospherix,) and exposed to chronic hypoxia (FIO2 = 0.10, 90% nitrogen) for 21 days under normobaric conditions. Rats maintained in room air served as controls. Some Rats were returned to room air on day 22, and maintained in normoxia for an additional 14 days. For rat studies animals were treated with daily subcutaneous injections of 10^5^ IU human recombinant interferon alpha 2b. This dose was chosen to approximately match the dose given to mice. The interferon was prepared and stored as described above for the chronic hypoxia mouse model.

### Right Ventricular Systolic Pressure

Right ventricular systolic pressure (RVSP) was measured essentially as described [20]. Briefly, mice or rats were anesthetized with sodium pentobarbital (60 mg/kg i.p. mice; 40 mg/kg i.p. rats) and ventilated via tracheotomy with room air. Body temperature was monitored and regulated with a rectal temperature probe and heating pad. RVSP was determined by placing a 1 F solid-state pressure-transducing catheter (Millar Instruments, Houston, TX, USA) directly into the right ventricle (RV). Data were acquired using a PowerLab data acquisition system and LabChart Pro software (AD Instruments).

### Right Ventricular Hypertrophy

Following hemodynamic measurements the vasculature was flushed with PBS, the heart was excised and right heart hypertrophy was determined by the ratio of the weight of the RV to the left ventricle (LV) plus septum (Fulton index) or the ration of weight of the RV to body weight. The right lung was tied off, dissected and flash frozen, and the left lung was perfused with paraformaldehyde (4%) for embedding in paraffin.

### Assessment of Pulmonary Vascular Remodeling

For mice, pulmonary vascular remodeling was assessed by counting the number of partially and fully muscularized peripheral arterioles (35–100 mm) per high-power field (200× total magnification). For each mouse, at least 20 high-power fields were analyzed in multiple lung sections. Wall thickness % was determined by measuring the thickness at four points on pulmonary arterioles using the Java-based image-processing program ImageJ (National Institutes of Health, Bethesda, MD, USA). Vascular occlusion was assessed in a blinded fashion by grading at least 50 small (<50 µm) pulmonary arterioles in at least 3 lung tissue samples per group.

### Serum IFNα

Serum IFNα was measured using commercially available ELISA kits ((R&D Systems, Minneapolis, MN).

### Immunohistochemistry

Paraffin-embedded lung sections (5 µm) were baked 60 min at 55°C, deparaffinized in xylenes and rehydrated through decreasing alcohol concentrations (three xylenes, 2×100%, 1×95%, 1×90%, 1×70% ethanol, 1×PBS, for 3 min each) followed by antigen retrieval citrate buffer by using a microwave. Smooth muscle α-actin staining was performed as described [21]. TUNNEL staining was performed using the Chemicon kit (S7100) using AEC (Vector) as color reagent and slides were counterstained using hematoxylin. PCNA (sc-7907, Santa Cruz) staining was done using the Elite Vectastain ABC kit (rabbit igG PK-6101) with DAB to obtain a color reaction.

### Cultured Cells

Control Human pulmonary artery endothelial cells (HPAECs) and human pulmonary artery smooth muscle cells (HPASMC) were from Lonza. Control and IPAH HPAEC were cultured in EBM2 media and HPASMC were cultured in SBM2 (Lonza) containing the recommended serum and growth factors. Cells were used between passages 4 and 9.

### Cell Proliferation

Briefly, HPAEC or HPASMC were serum-starved for 24 h in 12-well plates and treated with the indicated doses of IFNα with or without VEGF (50 ng/ml) or platelet-derived growth factor (PDGF) (10 ng/mL, Sigma P4056) for 24 h in the presence 0.2 µCi [3H] thymidine. Cell Proliferation was then determined by measuring [3H] incorporation as previously described [21].

### Western Blotting

30 µg of cell lysate was separated by SDS-PAGE and transferred to nitrocellulose membranes. Membranes were blocked in TBST (Tris-buffered saline, 0.1% Tween 20), 5% nonfat dry milk for 30 min, followed by incubation in primary antibody overnight. Membranes were washed in TBST before incubation for 1 h with horseradish peroxidase conjugated secondary antibodies. Membranes were washed and developed using enhanced chemiluminescence substrate (Pierce). Blots were probed against p21 (#sc-397 Santa Cruz), stat3 (#9132 Cell Signaling), phospho-Stat3 (9131 Cell Signaling), Stat1 (sc-98783 Santa Cruz), phospho-Stat1 (7649 Cell Signaling), Phospho-Akt (9271 Cell Signaling), Akt (61086 BD Transduction Laboratories), β-actin (4967 Cell Signaling).

### Statistical Analysis

Statistical analyses were performed by using Graphpad Prism software. Data were analyzed by one-way ANOVA and Tukey's post hoc tests. *P* values of <0.05 were considered significant.

## Results

### Treatment with IFNα improves hemodynamics in two animal models of PH

**Figure 1:**
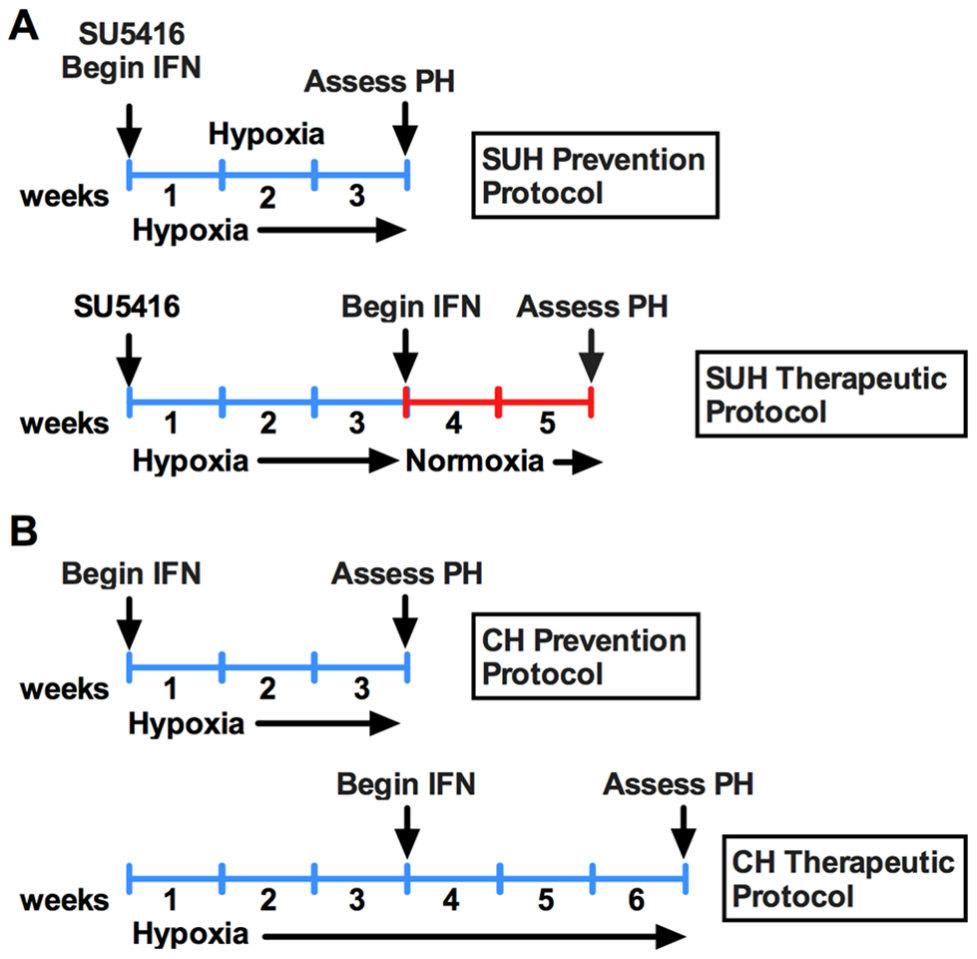
Schema of IFNα treatment protocols. (A) Schema of prevention and therapeutic protocols for IFNα treatment in SU5416/hypoxia-induced PH in rats. (B) Schema of prevention and therapeutic protocols for IFNα treatment in hypoxia-induced PH in mice.

**Figure 2:**
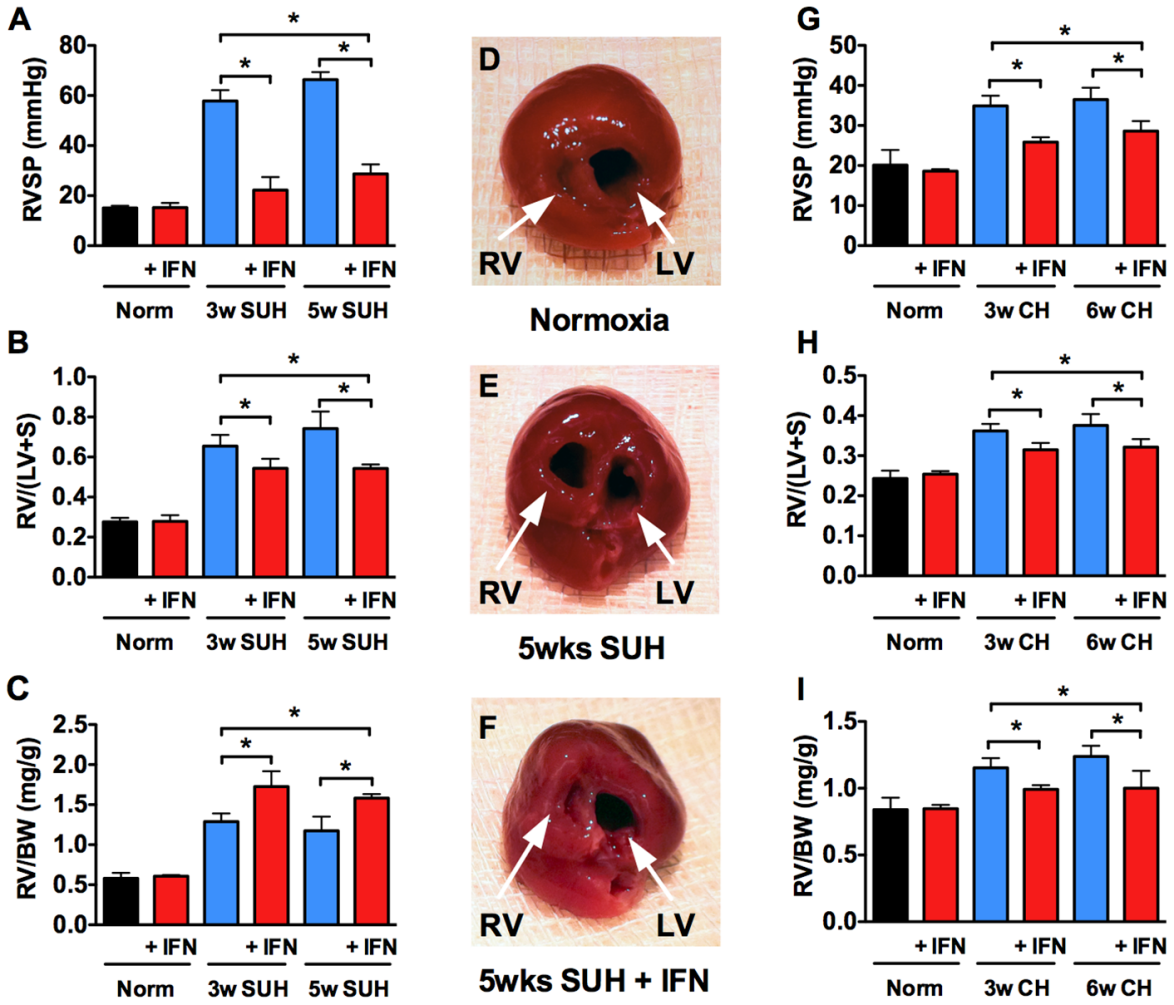
IFNα prevents and reverses experimental PH. (A) Effect of IFNα on RVSP and (B, C) RVH in SUH rats treated with IFNα or vehicle (n = 6 rats per group). (D–F) Representative Images of hearts from normoxic, 5 week SUH, and 5 week SUH rats treated with IFNα. Effect of IFNα on (G) RVSP and (H–I) RVH in hypoxic mice treated with IFNα or vehicle (n = 8 mice per group). Analysis of variance *P<0.05.

To examine the effect of IFNα on experimental PH we employed the rat model of SU5416/Hypoxia-induced PH (SUH). SUH rats were randomly assigned to a 3-week “prevention protocol” or a 5 week “therapeutic protocol” (**Fig. 1A**). In the prevention protocol, rats received a single injection of SU5416 (20 mg/kg s.c.) and were placed in hypoxia for 3 weeks (10% O_2_). These rats received daily injections of IFNα (10^5^ IU/day, s.c.) or sterile saline (vehicle) for the duration of the experiment. For the therapeutic protocol, the SUH rats were given a single injection of SU5416, exposed to 3-weeks of hypoxia and then returned to normoxia for 2 weeks. These rats were given daily injections IFNα (10^5^ IU/day, s.c.) or vehicle during the 2 week normoxic period. Rats maintained in normoxia served as controls. Treatment of SUH rats with IFNα using the prevention protocol attenuated the development of PH, as evidenced by decreased right ventricular systolic pressure (RVSP) and decreased right ventricular hypertrophy (RVH) compared to vehicle treated animals (**Fig. 2A** **–1C**). More importantly, IFNα treatment of SUH rats with established PH (therapeutic protocol) decreased RVSP and RVH compared with untreated SUH rats assessed for PH at 3 or 5 weeks (**Fig. 2A** **–1C**). Visual inspection of hearts from SUH rats further suggests that the hearts from 5-week SUH rats demonstrate increased RV dilatation compared with hearts from 3-weeks SUH rats, which was prevented by therapeutic IFNα (**Fig. 2D–F**).

To further explore the effect of IFNα in PH we also utilized the mouse model of hypoxia-induced PH. Mice were exposed to hypoxia for 3 weeks with or without concomitant IFNα (10^4^ I.U./day, s.c.). To establish the efficacy of IFNα on established disease, mice were exposed to 6 weeks of hypoxia and treated daily with IFNα (10^4^ I.U./day, s.c.) from week 4 through week 6 (**Figure 1B**). Mice maintained in normoxia served as controls. Treatment of mice with IFNα using the prevention or therapeutic protocol resulted in decreased disease severity as assessed by measuring RVSP and RVH (**Fig. 2G–I**). Importantly, in the therapeutic protocol, IFNα treated mice exhibited improvement when compared with the 3-week hypoxic mice demonstrating disease reversal.

### Exogenous IFNα acts via the type I interferon receptor

**Figure 3:**
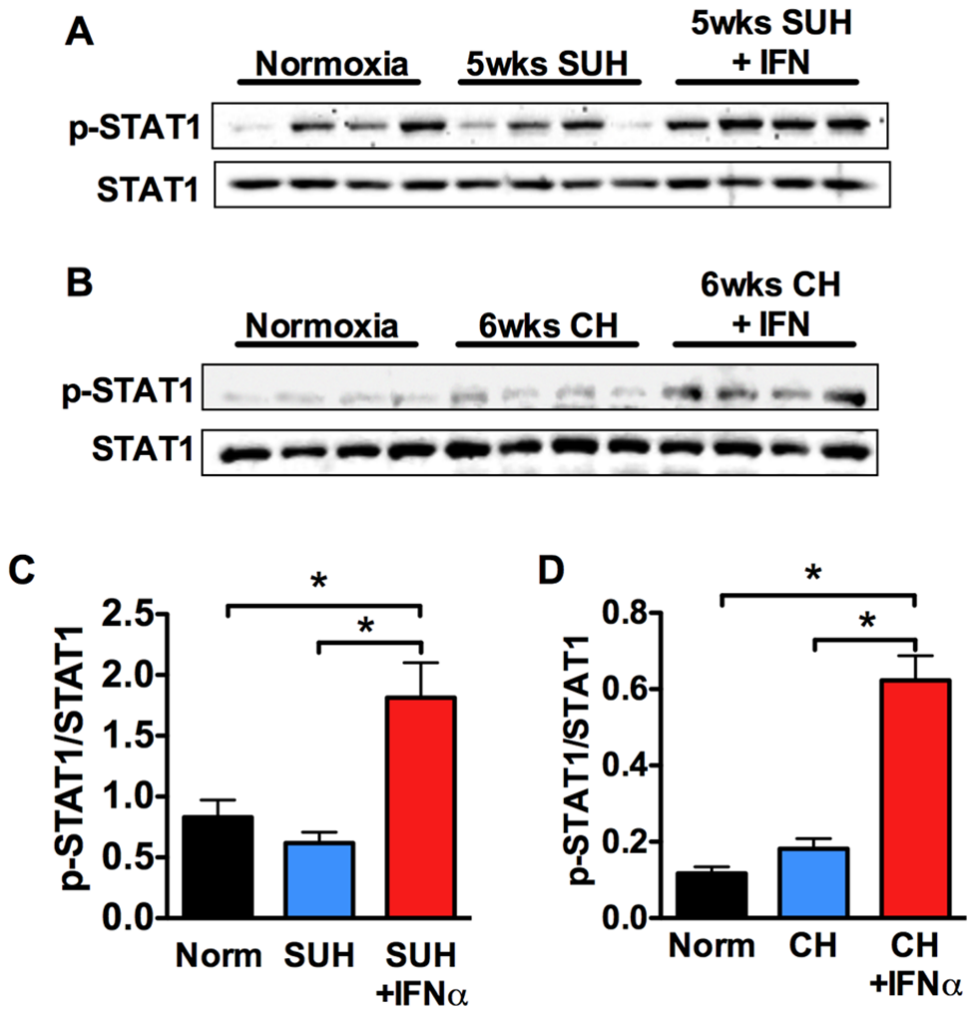
Human IFNα stimulates STAT1 phosphorylation in mice and rats. WB analysis of STAT1, phospho-STAT1 in whole lung homogenates from: (A) normoxic rats, 5 week SUH rats, and 5 week SUH rats treated with IFNα (n = 4 rats per group); or (B) normoxic mice, 6 week hypoxic mice, and 6 week hypoxic mice treated with IFNα. Densitometric ratio of phospho-STAT1 to STAT1 and phospho-STAT3 to STAT3 in lung tissue of different treatment groups in (C) SUH rats and (D) hypoxic mice.

Human recombinant IFNα exhibits reduced activity in rodents. To demonstrate that our results were not due to off-target effects of IFNα but occur via activation of the type I interferon receptor (IFNAR) we examined whether 1) human IFNα could elicit a typical type I interferon signaling response in rats and mice and 2) whether genetic deletion of a subunit of the type I interferon receptor could prevent the effect of IFNα in hypoxic mice. As expected of a type I IFN response, IFNα increased phosphorylation of STAT1 in both SUH rats (**Fig. 3A, C**) and hypoxic mice (**Fig. 3B, D**).

**Figure 4:**
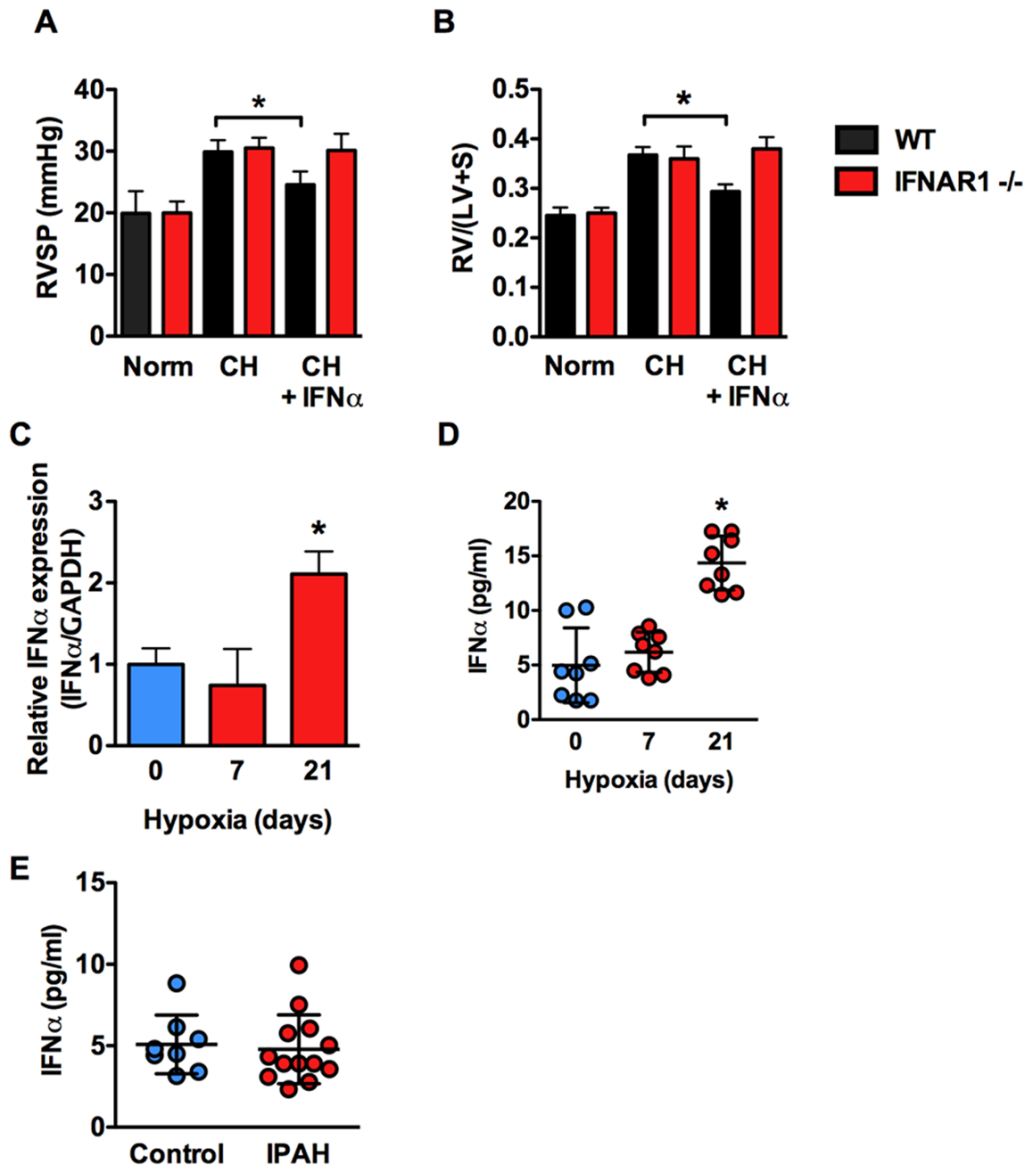
Human IFNα attenuates PH in mice in a IFNAR-dependent fashion. Effect of IFNα on (A) RVSP and (B) RVH in normoxic and hypoxic WT or IFNAR1 −/− mice (n = 6 mice per group). (C) Relative expression of IFNα (normalized to GAPDH) in total lung from C57BL/6J mice exposed to 0, 7, or 21 days of CH as determined by qRT-PCR. (D) Serum concentration of IFNα in C57BL/6J mice exposed to 0, 7, or 21 days CH as determined by ELISA. n = 8 animals per group. Analysis of variance *P<0.05. (E) Serum concentration of IFNα in control vs. IPAH human serum as determined by ELISA.

We next explored the effect of deleting the IFNAR1 subunit of the type I interferon receptor on the effect of IFNα in hypoxic mice. Deletion of this subunit abrogates type I interferon signaling in response to mouse IFNα. Exposure of WT or IFNAR1 −/− mice to 3-weeks hypoxia led to increased RVSP and RVH compared with normoxic controls (**Fig. 4A, B**). However, while treatment of WT mice with IFNα resulted in decreased RVSP and RVH, IFNα had no effect in IFNAR1 −/− mice demonstrating that human IFNα requires the type I interferon receptor in mice (**Fig. 4A, B**). These findings further demonstrate that endogenous IFNα does not affect disease development or progression in this model since there was no effect of IFNAR1 deletion on RVSP or RVH in hypoxic IFNAR1 −/− mice. This was despite the fact that IFNα mRNA in lung and circulating IFNα was elevated in CH mice after 21 days. We also determined the circulating levels of IFNα in control human (n = 8) vs. IPAH patient (n = 13) serum and found no difference.

### Treatment with IFNα regresses pulmonary vascular remodeling

**Figure 5:**
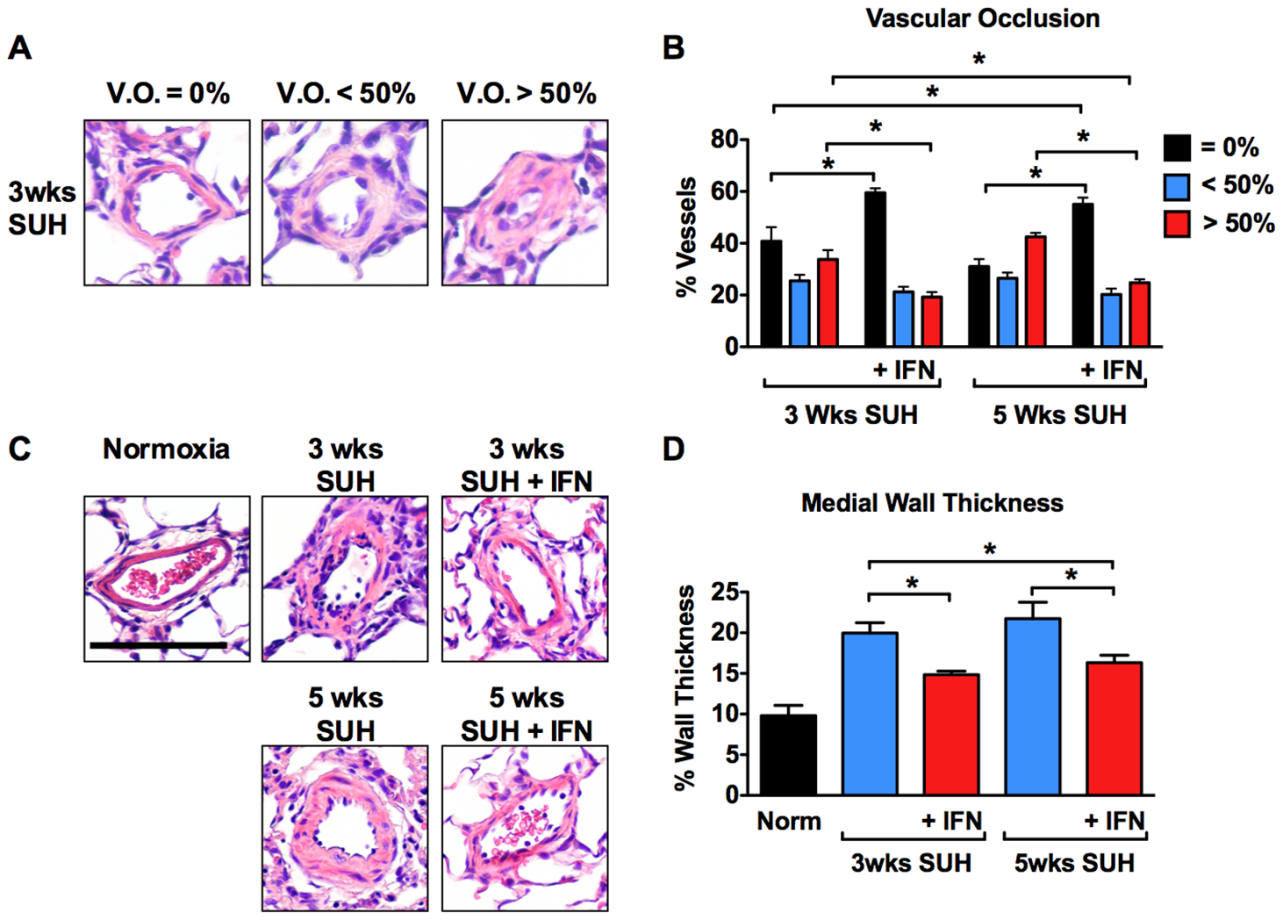
IFNα prevents and reverses pulmonary vascular remodeling in SUH rats. (A) Representative photomicrographs of small pulmonary arterioles (≤50 μm) from an SUH rat with vascular occlusion (V.O.) of 0%, <50%, and >50%. (B) Percent of small pulmonary arterioles (≤50 μm) with V.O. 0%, <50%, or >50% in SUH treatment groups (50 arterioles per animal, n = 4 animals per group). (C) Representative photomicrographs of pulmonary arterioles (≤100 μm) from SUH treatment groups demonstrating differences in wall thickness. (D) % Wall thickness in pulmonary arterioles (≤100 μm) from SUH treatment groups (20 arterioles per animal, n = 4 animals per group).

SU5416 with concurrent hypoxic exposure for 3 weeks caused severe PH with occlusive lesions in rats, which progressed in animals that were returned to normoxia for an additional two weeks. The proportion of vessels (≤50 um) that were occluded greater than 50% was less in the 3-week SUH or 5-week SUH treated with IFNα compared with untreated SUH rats (**Fig. 5A, B**). The 5-week SUH rats treated with IFNα also had a lower proportion of vessels that were occluded more than 50% when compared to 3-week SUH rats. This was associated with an increase in non-occluded vessels in IFNα treated vs. untreated SUH rats. Likewise, medial wall thickness of pulmonary arterioles (≤100 um) was less in the IFNα treated SUH rats and demonstrated reverse remodeling when comparing IFNα treated 5-week SUH rats to untreated 3-week SUH rats (**Fig. 5C** **,** **3D**).

### IFNα inhibits vascular cell proliferation *in vivo* and *in vitro*

**Figure 6:**
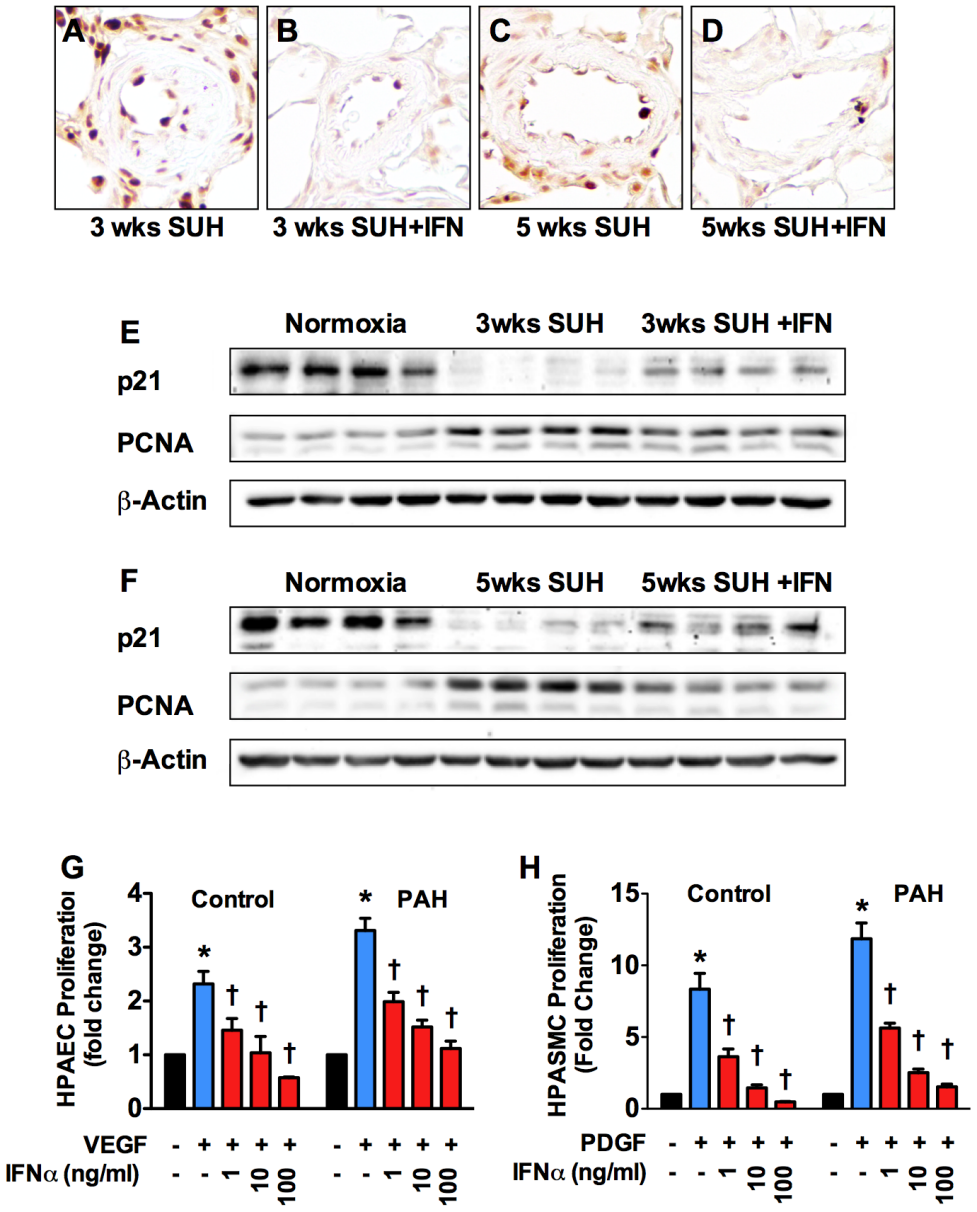
IFNα inhibits pulmonary vascular cell proliferation. (A–D) Representative 40x images of lung sections from 3 week SUH rat, 3 week SUH rat + IFNα, 5 weeks SUH rat, and 5 week SUH rats+ IFNα stained for PCNA (brown) as an indicator of proliferating cells. WB analysis for PCNA and p21 in whole lung lysates from (E) 3-week SUH rats or (F) 5-week SUH rats with or without IFNα (n = 4 animals per group). (G) Control or IPAH HPASMC were serum starved 24 h and then stimulated with PDGF (10 ng/ml) with or without increasing IFNα for 24 hours. (H) Control or IPAH HPAEC were serum starved overnight and then stimulated with VEGF (50 ng/ml) with or without increasing IFNα for 24 hours. Proliferation was assessed by measuring [H3]-thymidine incorporation. Analysis of variance *P<0.05.

Pulmonary vascular remodeling in SUH rats is characterized by increased proliferation of vascular smooth muscle and endothelial cells. Thus, we observed increased expression of proliferating cell nuclear antigen (PCNA) in the vessel wall of both 3 week and 5 week SUH rats. Consistent with the anti-proliferative effects of IFNα we observed less PCNA staining in the wall of pulmonary arterioles from SUH rats treated with IFNα (**Fig. 6A–D**). WB analysis confirmed the finding of increased PCNA in SUH rats and suppression of PCNA expression by IFNα (**Fig. 6E–F**). Furthermore, expression of the cyclin dependent kinase inhibitor p21 was decreased in SUH rats and IFNα increased p21 expression in SUH rats (**Fig. 6E–F**). Consistent with our finding of decreased number of proliferating cells in IFNα treated rats, IFNα dose-dependently inhibited the proliferation of human pulmonary artery smooth muscle cells (HPASMC) and human pulmonary artery endothelial cells (HPAEC) **(** **Fig. 6G–H** **) from both control and IPAH patients**.

### Decreased apoptotic cells in the pulmonary vascular wall of SUH rats treated with IFNα

**Figure 7:**
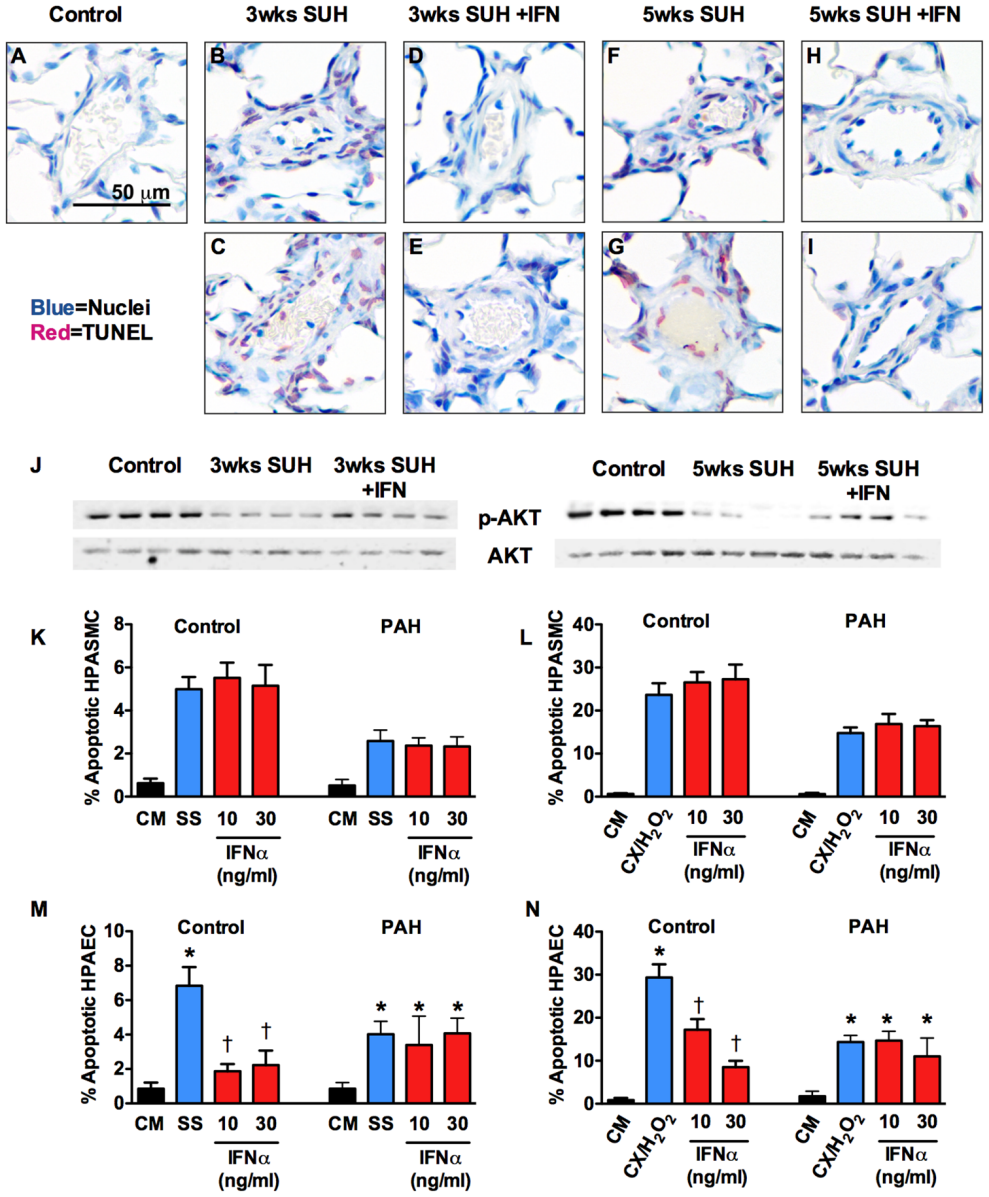
IFNα reduces the number of TUNEL positive cells in the pulmonary arterioles of SUH rats and inhibits HPAEC apoptosis. Representative photomicrographs of pulmonary arterioles stained for TUNEL (red) and nuclei (blue) in (A) normoxic control rats; (B, C) 3 week SUH rats; (D, E) 3 week SUH rats treated with IFNα; (F, G) 5 week SUH rats; and (H, I) 5 week SUH rats treated with IFNα. Photomicrographs are representative of 4–6 animals per group. (J) WB analysis for total AKT and phospho-AKT in whole lung lysates from 3-week SUH rats or 5-week SUH rats with or without IFNα (n = 4 animals per group). Control or IPAH HPASMC were grown in complete media and apoptosis was induced by (K) serum starvation or (L) cycloheximide plus hydrogen peroxide with or without IFNα. Control or IPAH HPAEC were grown in complete media and apoptosis was induced by (M) serum starvation or (N) cycloheximide plus hydrogen peroxide. Percent apoptotic cells was assessed by the ratio of TUNEL positive nuclei to total nuclei.

Because decreased proliferation can not fully explain our observation of reverse remodeling in IFNα treated SUH rats, we were interested in the effect of IFNα on pulmonary vascular cell apoptosis. There was increased number of TUNEL positive cells in the vessel wall of both 3 wk and 5 wk SUH rats when compared with normoxic controls. Treatment of SUH rats with IFNα caused a striking decrease in the number of TUNEL positive cells in the vessel wall using both the prevention and therapeutic protocol (**Fig. 7A–I**). Increased apoptosis in SUH rats was associated with decreased anti-apoptotic signaling as indicated by decreased AKT phosphorylation, which was reversed by IFNα treatment (**Fig. 7J**). In cultured cells, we found no effect of IFNα on HPASMC apoptosis (**Fig. 7K–L**) whereas IFNα potently inhibited apoptosis of control HPAEC, but not IPAH HPAEC, in response to serum starvation or the combination of cycloheximide and hydrogen peroxide (**Fig. 7M–N**).

## Discussion

IFNα belongs to a family of cytokines participating in innate immunity against viruses and other pathogens. IFNα also has anti-tumor activities due to its anti-proliferative, anti-angiogenic and immune-regulatory properties [22], [23]. Two isoforms of IFNα, IFNα 2a and IFNα 2b (used in this study), are used clinically for the treatment of Hepatitis B and C as well as various cancers. The seminal finding of this study is that IFNα 2b attenuates the onset of PH and more excitingly causes regression of established PH in two experimental animal models of the disease. Of particular interest are our findings that IFNα can cause regression of established PH in SUH rats since the resulting hemodynamic and histopathologic changes in this model most closely mimic those in human PH [24], [25].

In this study we demonstrate that, in two rodent models of PH, IFNα significantly reduced RVSP and RVH compared with vehicle-treated animals. Importantly, we also demonstrate that SUH rats or hypoxic mice treated with IFNα using our therapeutic protocol showed significant improvements in RVSP and RVH when compared with the 3 week control animals demonstrating reversal of established disease. The positive changes in hemodynamics and RVH were accompanied by decreased pulmonary vascular remodeling and perivascular inflammation. In the rat SUH model, treatment with IFNα led to a decrease in the number of occlusive lesions in both treated groups compared with vehicle. Importantly, in 5-week SUH rats we found less occlusive lesions when compared with SUH 3-week control animals. Similarly, we observed a decrease in medial wall thickness of SUH rats treated with IFNα, again with evidence of reverse remodeling in the 5-week SUH rats.

The effect of IFNα on pulmonary vascular remodeling was accompanied by reduced numbers of PCNA-positive cells in pulmonary arterioles from IFNα treated animals demonstrating decreased pulmonary vascular cell proliferation *in vivo*. *In vitro* experiments further demonstrated that IFNα directly inhibits proliferation of both HPAEC and HPASMC from control or IPAH patients. While the anti-proliferative effect of IFNα is sufficient to explain the suppression of pulmonary vascular remodeling in prevention groups, it cannot completely explain reverse remodeling in the SUH therapeutic groups.

This led us to explore the effect of IFNα on apoptosis. In our *in vitro* studies we found that while IFNα had no effect on control or IPAH HPASMC, IFNα inhibited apoptosis in control HPAEC but not in ECs from IPAH patients. As was previously demonstrated the IPAH HPAEC were somewhat resistant to apoptosis as compared to control [26], which may partially explain why IFNα had no effect on these cells. These results suggest that IFNα may prevent or attenuate apoptosis of healthy endothelium helping to preserve normal endothelial function. Despite these *in vitro* results demonstrating decreased EC apoptosis, it was somewhat surprising to find a striking decrease in TUNEL positive cells in the pulmonary vascular wall of SUH rats treated with IFNα. We had anticipated that reverse remodeling requires increased apoptosis. There are several possible explanations for these observations. Studies demonstrate that EC apoptosis contributes to pathologic remodeling in the SUH model of pulmonary hypertension and that caspase inhibition ameliorates PH in this model [27]. Thus, direct inhibition of EC apoptosis is likely to play a role in the therapeutic effects observed in response to IFNα. We also cannot rule out the possibility that in the therapeutic model there was an early increase in apoptosis in response to IFNα that resolved before endpoint measurements were made.

Another interesting possibility is the idea that phagocytosis of apoptotic cells (efferocytosis) is impaired in the SUH model and that IFNα stimulates efferocytosis. The number of apoptotic cells in a tissue is affected both by the rate of apoptosis and the rate of efferocytosis by macrophages and resident cells. Apoptotic cells that are not cleared become necrotic causing release of pro-inflammatory molecules [28]. Impaired efferocytosis is linked to the pathogenesis of chronic vascular and pulmonary inflammatory diseases including atherosclerosis [29], systemic lupus erythematosus, chronic obstructive pulmonary disease (COPD) [30], [31], cystic fibrosis [32] and asthma [33], [34]. Interestingly, in a 2006 review article Vandivier et al. reported that efferocytosis is impaired in a SU5416 model of COPD [28]. In addition, we recently demonstrated a role for high mobility group box 1 in the pathogenesis of PH [35], [36], and high mobility group box 1 inhibits efferocytosis [37]–[39]. In contrast, efferocytosis suppresses innate immunity and promotes its resolution by suppressing the expression of inflammatory mediators [40]. To that end, IFNα increases phagocytosis by macrophages [41], [42] suggesting that the effect of IFNα in SUH rats may be partially attributable to stimulation of efferocytosis. Additional studies are needed to address the role of apoptosis and efferocytosis in the effect of IFNα on PH and in the pathogenesis of the disease itself.

IFNα is a pleiotropic cytokine that affects many cell types. Thus, it is likely that other cell types beyond those explored in this study are involved in the effect of IFNα in these models of PH. Of interest is the possibility that the effects of IFNα may be mediated by activation of natural killer cells. Ormiston et al. recently demonstrated impairment of natural killer cell phenotype and function in PH patients, hypoxia-induced PH in mice, and monocrotaline-induced PH in rats [43]. In contrast, IFNα augments natural killer cell cytotoxicity and up-regulates expression of cytolytic effectors Fas-L and perforin [44], [45]. It is tempting to hypothesize that the effect IFNα has on pulmonary vascular remodeling is partly due to increased natural killer cell function.

Despite our finding that IFNα prevents and reverses experimental pulmonary hypertension in 2 distinct models, chronic treatment with IFNα for hepatitis C or chronic myelogenous leukemia was associated with the onset of PH in humans and the Food and Drug Administration has labeled IFNα with a warning about the risk of PH with its use. Recently, Dhillon et al. [12] reported four cases of PH in hepatitis C patients treated with IFN-α. Two of the patients were non-cirrhotic and two were post-liver transplant. Other causes of PH including portopulmonary hypertension and were ruled out. The authors suggest the acceleration of a previously subclinical phenomenon caused by factors such as human herpes virus 8, hepatitis C virus, or genetic predisposition [12] as potential mechanisms. Another case report described reversible PH in a chronic myelogenous leukemia patient treated with IFNα. Interestingly, there are several case reports of chronic myelogenous leukemia patients developing PH after treatment with the tyrosine kinase inhibitor dasatinib [46]–[48] and, like IFNα, dasatinib has been shown to reverse experimental PH [49]. The fact that these two unrelated drugs lead to the development of PH in chronic myelogenous leukemia, but reverse experimental PH suggests that some feature of chronic myelogenous leukemia renders a fraction of these patients susceptible to PH. In either case, PH remains a rare complication of these drugs suggesting individual and/or disease-related susceptibility to PH.

In terms of a role for endogenous IFNα in the development of PH we found that despite a slight elevation in circulating levels of IFNα in mice after 21 days of CH that deletion of IFNAR1 subunit of the type I interferon receptor had no effect on the progression of PH. Interestingly this is different from what was recently reported by George et al. where they found that deletion of this subunit protected mice from the development of chronic hypoxia-induced PH. In this study they also report that IFNAR1 is upregulated in Systemic Sclerosis patients with PH and that Interferon Regulated Protein 10 (IP10) correlated positively with pulmonary hemodynamics and serum brain natriuretic peptide and negatively with 6-minute walk test and cardiac index [50]. The correlation of IP10 with PH in SSC patients has also been shown by another group, however, that group and another failed to find a direct correlation between circulating IFNα and PH in SSC patients [51], [52]. We also found no evidence for increased circulating IFNα in a limited number of IPAH patients.

To our knowledge this is only the second demonstration of disease reversal in the SUH rat model of PH. While case studies suggest that IFNα therapy may cause pulmonary hypertension, our data raise legitimate questions as to the role of IFNα in this process. IFNα is a pleiotropic cytokine that mediates a wide range of biological effects including, anti-proliferative, anti-angiogenic and anti-tumor activities, which theoretically could provide benefit to PH patients. The reversal of PH with an immunotherapeutic modality is novel and provides proof of principle that immunotherapy can have a positive impact on PH progression. While evidence of IFNα-induced PH in humans might mean that IFNα will never be used to treat human PH our results warrant further investigation of IFNα and other immunotherapeutics in PH.
